# The Role of Nonophthalmologists in the Primary Evaluation of Head Injury Patients with Ocular Injuries

**DOI:** 10.3390/jpm11111220

**Published:** 2021-11-18

**Authors:** Chen-Hua Lin, Xiao Chun Ling, Wei-Chi Wu, Kuan-Jen Chen, Chi-Hsun Hsieh, Chien-Hung Liao, Chih-Yuan Fu

**Affiliations:** 1 Department of Trauma and Emergency Surgery, Chang Gung Memorial Hospital, Chang Gung University, Taoyuan 333, Taiwan; wilmak0209@gmail.com (C.-H.L.); hsiehchihsun@yahoo.com.tw (C.-H.H.); m7077@cgmh.org.tw (C.-H.L.); 2 Department of Ophthalmology, Taipei Tzu Chi Hospital, The Buddhist Tzu Chi Medical Foundation, New Taipei City 231, Taiwan; 3 Department of Ophthalmology, Chang Gung Memorial Hospital, Chang Gung University, Taoyuan 333, Taiwan; lingxiaochun1991@gmail.com (X.C.L.); weichi666@gmail.com (W.-C.W.); cgr999@gmail.com (K.-J.C.)

**Keywords:** head trauma, ocular injury, ophthalmologist, consultation

## Abstract

Purpose—Visual complaints are common in trauma cases. However, not every institution provides immediate ophthalmic consultations 24 h per day. Some patients may receive an ophthalmic consultation but without positive findings. We tried to evaluate risk factors for ocular emergencies in trauma patients. Then, the ophthalmologists could be selectively consulted. Methods—From January 2019 to December 2019, head injuries patients concurrent with suspected ocular injuries were retrospectively reviewed. All of the patients received comprehensive ophthalmic examinations by ophthalmologists. Patients with and without ocular injuries were compared. Specific ophthalmic evaluations that could be primarily performed by primary trauma surgeons were also analyzed in detail. Results—One hundred forty cases were studied. Eighty-nine (63.6%) patients had ocular lesions on computed tomography (CT) scans or needed ophthalmic medical/surgical intervention. Near 70% (69.7%, 62/89) of patients with ocular injuries were diagnosed by CT scans. There was a significantly higher proportion of penetrating injuries in patients with ocular injuries than in patients without ocular injuries (22.5% vs. 3.9%, *p* = 0.004). Among the patients with blunt injuries (*N* = 118), 69 (58.5%) patients had ocular injuries. These patients had significantly higher proportions of periorbital swelling (89.9% vs. 67.3%, *p* = 0.002) and diplopia (26.1% vs. 8.2%, *p* = 0.014) than patients without ocular injuries. Conclusions—In patients with head injuries, concomitant ocular injuries with indications for referral should always be considered. CT serves as a rapid and essential diagnostic tool for the evaluation of concomitant ocular injuries. Ophthalmologists could be selectively consulted for patients with penetrating injuries or specific ocular presentations, thus reducing the burden of ophthalmologists.

## 1. Introduction

Visual complaints are common in trauma cases [1]. A previous study reported that 74% of head injury patients had visual disturbance and confirmed visual impairment occurred in 38% of all cases [2]. Traumatic injuries to the eye (either blunt or penetrating types) may cause vision-threatening damage, such as globe rupture, orbital compartment syndrome, retinal detachment, or traumatic optic neuropathy [3]. One study demonstrated that accommodative dysfunction, convergence insufficiency, and visual field loss are common sequelae of head injury [4]. Therefore, a systematic ophthalmic history and clinical examinations are needed for the evaluation of head injury patients in the emergency department.

Although ocular examinations can be primarily conducted by emergency department physicians or primary trauma surgeons, there are still limitations with regard to specialized ophthalmic exams and surgical interventions [5]. However, not every institution provides immediate ophthalmic consultations 24 h per day. Studies have shown that ophthalmologists tend to discover injuries that are not recognized by nonophthalmologists, with overall discovery rates of 26% and 2~23%, respectively [5,6]. Thus, early identification of ocular emergencies and immediate referral to a medical institution with an available ophthalmologist are necessary.

In this study, head injury patients were retrospectively reviewed to evaluate the characteristics of such patients with concomitant ocular trauma. We hypothesized that ophthalmologists could be selectively consulted for head injury cases with risk factors for ocular emergencies. Furthermore, the roles of first-line trauma surgeons in the management of ocular injury cases was also discussed.

## 2. Material and Methods

### 2.1. Study Design and Setting

From January 2019 to December 2019, the data of patients with head injuries concurrent with suspected ocular injuries who visited the emergency department of our institution were reviewed retrospectively. Our institution serves as a level I trauma referral center with more than 3000 beds and covers a population of more than 5,000,000 people in adjacent counties. In our institution, a board-certified ophthalmology consultant is available for consultation within one hour for 24 h per day. Additionally, surgeries for ocular emergencies can be performed by ophthalmologists almost immediately when indicated.

Head injury patients in our emergency department were treated according to an established protocol based on the advanced trauma life support guidelines and evaluated by primary trauma surgeons [7]. After primary assessment and resuscitation, a computed tomography (CT) scan was performed for the evaluation of head injuries. In patients with subjective or objective lesions which were clinically suspected as ocular injuries, such as diplopia, periorbital swelling, or decrease in visual acuity, the ophthalmologist was contacted for a comprehensive evaluation. Subsequent treatments for ocular injuries were then performed accordingly.

### 2.2. Definitions

In the current study, patients with ocular injuries were defined as patients with ocular lesions on CT scans and patients who needed ophthalmic medical/surgical intervention.

### 2.3. Data Collection

Head injury patients with and without concomitant ocular injuries were compared. Patients’ general demographics, trauma mechanisms, and associated symptoms were recorded and analyzed. Specific ophthalmic evaluations that could be primarily performed by emergency department physicians were also analyzed in detail. The ocular trauma score was applied to categorize the patients into groups according to visual outcomes, and the distribution of patients in each group was also discussed [8].

### 2.4. Statistical Analysis

In the present study, nominal data are presented as percentages and were compared using a Chi-square test, whereas numerical data are presented as medians and interquartile ranges and were compared using the Mann–Whitney U test. All statistical analyses were performed using the SPSS^TM^ (Statistical Package for the Social Sciences, Chicago, IL, USA) (version 24.0). A *p* value < 0.05 was considered statistically significant.

## 3. Results

**Figure 1:**
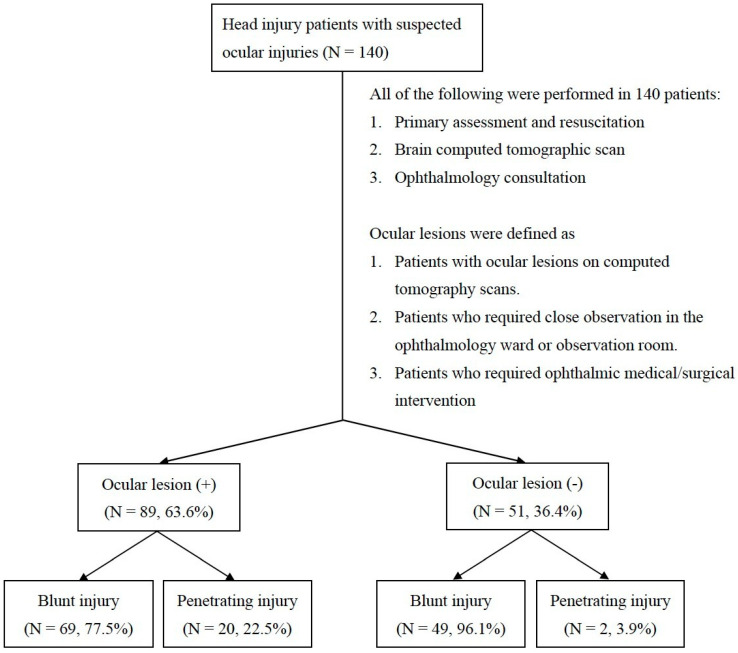
A total of 140 head injury patients underwent primary assessment, resuscitation, brain CT and ophthalmology consultation in the emergency department. Eighty-nine patients were defined as ocular lesion (+), and 51 patients were defined as ocular lesion (−). In each group, patients were subcategorized according to the mechanism of injury (either blunt or penetrating injury).

During the study period, 550 patients had head injuries. One hundred forty of them had suspected ocular injuries and underwent comprehensive ophthalmic evaluation in the emergency department. Eighty-nine patients (63.6%) had ocular lesions on CT scans or needed ophthalmic medical/surgical intervention. In contrast, 36.4% of patients had negative ophthalmic evaluations (Figure 1).

Table 1 shows comparisons between the patients with and without ocular injuries. There was a significantly higher proportion of penetrating injuries in the patients with ocular injuries than in the patients without ocular injuries (22.5% vs. 3.9%, *p* = 0.004). There was no significant difference in the general demographics (age, sex), laterality, or associated symptoms between the patients with and without ocular injuries.

For patients with blunt injuries, Table 2 shows comparisons of primary ocular examination results, which could be evaluated by emergency department physicians, between patients with and without ocular injuries (*N* = 118). Of these patients, 69 (58.5%) had ocular injuries. Patients with ocular injuries had a significantly higher proportion of periorbital swelling (89.9% vs. 67.3%, *p* = 0.002) and a significantly higher proportion of diplopia (26.1% vs. 8.2%, *p* = 0.014) than patients without ocular injuries.

In the current study, 69.7% (62/89) of patients with ocular injuries were diagnosed with CT scans. The other 30.3% of ocular injury patients without ocular lesions on CT scans were evaluated and analyzed (Table 3). Compared with patients without ocular injuries, patients with ocular injuries had significantly more penetrating injuries (70.4% vs. 3.9%, *p* < 0.001), more hyphema (51.9% vs. 25.5%, *p* = 0.038) and more uveal deformities (51.9% vs. 0.0%, *p* < 0.001).

**Figure 2:**
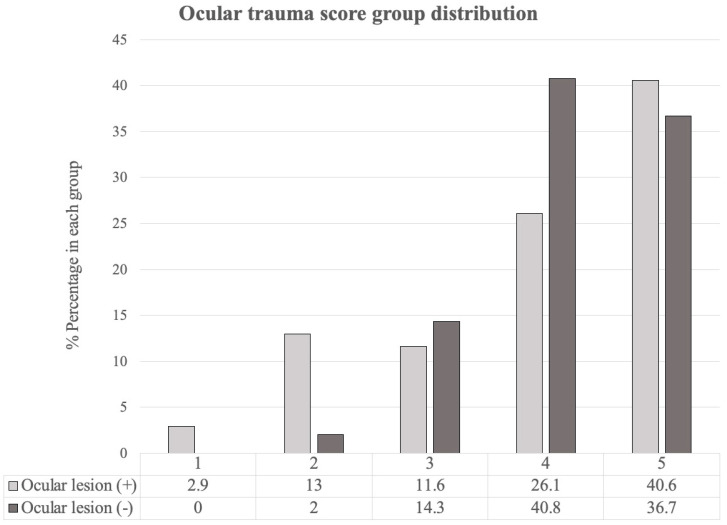
Distribution of the ocular trauma score in blunt head injury patients.

The ocular trauma score was applied to categorize blunt head injury patients (Figure 2). We found that most patients were distributed in groups 4 and 5, with proportions of 66.9% in patients with ocular emergencies and 77.3% in patients without ocular emergencies.

## 4. Discussion

Head injury patients often have concomitant ocular injuries [9]. However, not every medical institution provides immediate consultations by an ophthalmologist 24 h per day. Some ocular emergencies should be treated promptly lest irreversible changes may occur. Thus, early detection of ocular emergencies is mandatory in the evaluation of head trauma patients. We believe the results of this study can help guide first-line responders in accurately evaluating as well as effectively managing concomitant ocular injuries in head trauma patients.

Ocular trauma in the emergency department could be roughly classified according to the mechanism of injury, such as penetrating or blunt trauma. In our study, 77.5% of the patients who needed an ophthalmic consultation had blunt injuries, while 22.5% had penetrating injuries. However, among all the penetrating injury patients, 90.9% were considered to have ocular emergencies and referred to an ophthalmologist. A penetrating injury from high-velocity trauma or a sharp object may result in globe rupture, extravasation of intraocular content, and a retained intraocular foreign body. Care should be taken by emergency department physicians to decrease iatrogenic increases in intraocular pressure while examining the orbit [10]. Severe vision loss may result from mechanical injury or from posttraumatic complications such as endophthalmitis, retinal detachment, metal toxicity or sympathetic ophthalmia [11]. Penetrating head trauma could be an important factor in the evaluation of ocular emergencies for primary physicians due to the easily identified yet problematic nature of the mechanism. An early consultation with an ophthalmologist is suggested, and early transfer to institutions with ophthalmologists should be considered.

Furthermore, experience has shown that field triage of trauma victims with ocular emergencies is extremely difficult. Under-triage rates are as high as 70% among patients initially sent to inappropriate medical centers [12]. Inter-facility transfers of incorrectly triaged patients to trauma centers with subspecialties are frequently required for these patients. This results in additional morbidity in the patient and a financial burden for the healthcare system [13]. Therefore, accurate triage criteria for transport to the appropriate level trauma center are important. The penetrating injury is an easily-identified injury with high probability of associated ocular emergencies. A triage to trauma centers with ophthalmologists is suggested for these patients per the results of current study.

Not all trauma surgeons are familiar with specific ocular examinations which may need subspecialty training or instruments. Therefore, simple and easily performed physical examinations are important for first-line trauma surgeons who need to evaluate ocular emergencies. Juang et al. developed a guideline to assist emergency department physicians in ophthalmic evaluations [14]. The clinical assessment should include history taking, a visual acuity evaluation, a pupil examination (including assessing relative afferent pupillary defects), an external examination, an extraocular movement assessment, a visual field assessment, and a color vision assessment. Since penetrating ocular trauma usually requires an urgent evaluation by an ophthalmologist, patients with blunt trauma who have concomitant ocular emergencies should be evaluated carefully by a trauma surgeon. Among all the blunt trauma patients, we found that diplopia and periorbital swelling were the most common symptoms in ocular emergency cases. Traumatic diplopia may be the result of injury along the visual pathway, including the internal structures of the eye; extraocular muscles; optic nerves; and third, fourth, and sixth cranial nerves [15,16]. Blunt ocular trauma commonly causes orbital soft tissue swelling, orbital wall fractures, extraocular muscle prolapse, and traumatic optic neuropathy, all of which may present as diplopia. Additional attention should be paid in the emergency department when blunt trauma patients have these visual manifestations.

In patients with head injuries, thorough ocular examinations for the detection of ocular injuries may be difficult due to consciousness disturbance and associated trauma to the face and head. Thus, CT imaging is valuable in the initial assessment by trauma surgeons. A large retrospective case series that analyzed the use of CT imaging in the emergency department for undifferentiated eye complaints revealed that 68% of patients with triage complaints of ocular trauma who underwent CT imaging of the brain had positive ocular imaging findings, such as orbital wall fractures and globe rupture [17,18]. CT scans have a sensitivity of 64–78% for detecting orbital wall fractures and a sensitivity of 75% for detecting global rupture [19].

Regarding intraocular foreign bodies, CT is currently considered the gold standard for the detection, localization and characterization of both metallic and nonmetallic intraocular foreign bodies. Magnetic resonance imaging may be difficult to perform in emergency situations, and it is contraindicated if there is a possibility that a metallic intraocular foreign body is present [20]. In the current study, near 70% of ocular injuries were evaluated with CT. Therefore, CT plays an important role in the evaluation of patients with head injuries concomitant with ocular injuries.

In situations where CT imaging was grossly “normal”, we found that hyphema (accumulation of blood in the anterior chamber of the eye) and uveal deformity warranted a prompt comprehensive eye examination by an ophthalmologist. This result emphasizes the importance of inspecting the pupil and anatomic structures of the anterior portion of the eye by trauma surgeons.

In our study, we used the ocular trauma score to categorize patients suffering from blunt injury. The ocular trauma score is used to predict the visual outcomes of patients after ocular trauma, ranging from 1 (most severe injury and worst prognosis at 6 months) to 5 (least severe injury and best possible prognosis at 6 months). Each score is associated with a range of predicted post-injury visual acuities [8]. We found that most blunt trauma patients in both injury groups had “less severe” (groups 4 and 5) injuries using the ocular trauma score classification, with percentages of 66.7% and 77.5% in each group, respectively. A total of 15.9% of blunt trauma patients who needed ophthalmic consultations were classified into group 1 and group 2. The distribution of patients in each group indicated that most blunt ocular trauma cases were not severe and that there was a higher proportion of severe injury in the group with ocular emergencies than in the group without ocular emergencies. Primary trauma surgeons can use the ocular trauma score as guidance for referral to an ophthalmologist and to manage the expectations of patients regarding visual prognosis.

The limitations of this study include its retrospective design and limited patient sample from a single institution. In addition, we understand that there must be patients who suffered from ocular injuries but were diagnosed at a later stage. However, the patient number could be very small because of strict patient selection criteria of the current study (only one patient among these 140 patients). Possible selection bias may have limited our conclusions. Another concern is that some indications for ophthalmologist consultations were difficult to define, making it challenging to determine whether they should be included in this study. For instance, nonspecific ocular swelling or pain may result in confusion when trauma surgeons are evaluating the need for referral. Furthermore, some consultations may be performed at the request of the patient or even for medical-legal reasons. Nevertheless, the results of the current study provide primary trauma surgeons an evidence-based guide to identify head injury patients with concomitant ocular emergencies. Patients with specific characteristics should be triaged to institutions with ophthalmologists, and thus unnecessary transfer among institutions could be avoided. Further studies with larger sample sizes and prospective designs are needed to establish even more accurate protocols for ophthalmologist consultations.

## 5. Conclusions

In patients with head injuries, concomitant ocular injuries with indications for referral should always be considered. CT serves as a rapid and essential diagnostic tool for the evaluation of concomitant ocular injuries. Ophthalmologists could be selectively consulted for patients with certain injury mechanisms and ocular presentations, thus reducing the burden of ophthalmologists.

## Figures and Tables

**Table 1:** Characteristics of head injury patients who underwent an ophthalmology consultation in the emergency department (*N* = 140).

	Ocular Lesion (+) (*N* = 89)	Ocular Lesion (−) (*N* = 51)	*p*-Value
Age (years)	45 (27)	50 (36)	0.170 *
Sex (*N*, %)			0.611 ^†^
Male	63 (70.8%)	34 (66.7%)	
Female	26 (29.2%)	17 (33.3%)	
Laterality (*N*, %)			0.537 ^†^
OD	34 (38.2%)	23 (45.1%)	
OS	47 (52.8%)	22 (43.1%)	
OU	8 (9.0%)	6 (11.8%)	
Trauma mechanism (*N*, %)			0.004 ^†^
Penetrating	20 (22.5%)	2 (3.9%)	
Blunt	69 (77.5%)	49 (96.1%)	
Associated symptoms (*N*, %)			
Initial loss of consciousness	26 (29.2%)	12 (23.5%)	0.467 ^†^
Altered mental status	4 (4.5%)	4 (7.8%)	0.411 ^†^
Posttraumatic amnesia	14 (15.7%)	10 (19.6%)	0.558 ^†^
Dizziness	6 (6.7%)	5 (9.8%)	0.517 ^†^
Nausea	5 (5.6%)	2 (3.9%)	0.658 ^†^
Vomiting	5 (5.6%)	0 (0.0%)	0.085 ^†^
Headache	6 (6.7%)	4 (7.8%)	0.808 ^†^
Facial wounds	41 (46.1%)	22 (43.1%)	0.737 ^†^

Numerical data: median (interquartile range); Nominal data: *N* (percentage). * Mann–Whitney U test. ^†^ Chi-square test. Each patient could have had more than one positive sign. Thus, the total number of positive signs exceeds the total number of patients. Ocular lesion = 1. Retrobulbar hemorrhage, ocular muscle incarceration or orbital wall fractures on the CT scan. 2. Patients who required close observation in the ophthalmology ward or observation room. 3. Patients who required ophthalmic surgical intervention/exploration. Abbreviations: OD: Right eye; OS: Left eye; OU: Bilateral eyes

**Table 2:** Results of primary eye examinations in the emergency department in blunt head injury patients and comparisons between patients with and without positive ophthalmic lesions (*N* = 118).

Primary Eye Examination in the Emergency Department	Ocular Lesion (+) (*N* = 69, 58.5%)	Ocular Lesion (−) (*N* = 49, 41.5%)	*p*-Value ^†^
Visual loss	4 (5.8%)	1 (2.0%)	0.318
Blurred vision	37 (53.6%)	26 (53.1%)	0.952
Diplopia	18 (26.1%)	4 (8.2%)	0.014
Extraocular movement limitation	19 (27.5%)	7 (14.3%)	0.087
Relative afferent pupillary defect	7 (10.1%)	3 (6.1%)	0.439
Periorbital swelling	62 (89.9%)	33 (67.3%)	0.002
Lid laceration	18 (26.1%)	10 (20.4%)	0.475
Hyphema	19 (27.5%)	13 (26.5%)	0.904
Uveal deformities	4 (5.8%)	0 (0.0%)	0.086

Nominal data: *N* (percentage). ^†^ Chi-square test. Each patient could have had more than one positive sign. Thus, the total number of positive signs exceeds the total number of patients. Ocular lesion = 1. Retrobulbar hemorrhage, ocular muscle incarceration or orbital wall fractures on the CT scan. 2. Patients who required close observation in the ophthalmology ward or observation room. 3. Patients who required ophthalmic surgical intervention/exploration.

**Table 3:** Characteristics of head injury patients who underwent an ophthalmology consultation in the emergency department and without ocular lesions on CT scans (*N* = 78).

	Ocular Lesion (+) (*N* = 27)	Ocular Lesion (−) (*N* = 51)	*p*-Value
Age (years)	52 (15)	50 (36)	0.950 *
Sex (*N*, %)			0.011 ^†^
Male	25 (92.6%)	34 (66.7%)	
Female	2 (7.4%)	17 (33.3%)	
Laterality (*N*, %)			0.196 ^†^
OD	9 (33.3%)	23 (45.1%)	
OS	17 (63.0%)	22 (43.1%)	
OU	1 (3.7%)	6 (11.8%)	
Type of injury (*N*, %)			<0.001 ^†^
Penetrating	19 (70.4%)	2 (3.9%)	
Blunt	8 (29.6%)	49 (96.1%)	
Associated symptoms (*N*, %)			
Initial loss of consciousness	1 (3.7%)	12 (23.5%)	0.055 ^†^
Altered mental status	0 (0.0%)	4 (7.8%)	0.340 ^†^
Posttraumatic amnesia	1 (3.7%)	10 (19.6%)	0.115 ^†^
Dizziness	0 (0.0%)	5 (9.8%)	0.232 ^†^
Nausea	0 (0.0%)	2 (3.9%)	0.772 ^†^
Headache	1 (3.7%)	4 (7.8%)	0.823 ^†^
Facial wounds	5 (18.5%)	22 (43.1%)	0.054 ^†^
Visual loss	2 (7.4%)	1 (2.0%)	0.568 ^†^
Blurred vision	21 (77.8%)	27 (52.9%)	0.057 ^†^
Diplopia	0 (0.0%)	4 (7.8%)	0.340 ^†^
Extraocular movement limitation	1 (3.7%)	7 (13.7%)	0.319 ^†^
Relative afferent pupillary defect	0 (0.0%)	3 (5.9%)	0.505 ^†^
Lid laceration	7 (25.9%)	10 (19.6%)	0.723 ^†^
Hyphema	14 (51.9%)	13 (25.5%)	0.038 ^†^
Uveal deformities	14 (51.9%)	0 (0.0%)	<0.001 ^†^

Numerical data: median (interquartile range); Nominal data: *N* (percentage). * Mann–Whitney U ^†^ Chi-square test. Each patient could have had more than one positive sign. Thus, the total number of positive signs exceeds the total number of patients. Ocular lesion = 1. Patients who needed close observation in the ophthalmology ward or observation room. 2. Patients who required ophthalmologic surgical intervention/exploration. Abbreviations: OD: Right eye; OS: Left eye; OU: Bilateral eyes

## Data Availability

Availability of supporting data: Please contact author for data requests. (Fu, Chih-Yuan, E-mail: drfu5564@gmail.com).
